# Applying Systems Engineering Reduces Radiology Transport Cycle Times in the Emergency Department

**DOI:** 10.5811/westjem.2016.12.32457

**Published:** 2017-02-21

**Authors:** Benjamin A. White, Brian J. Yun, Michael H. Lev, Ali S. Raja

**Affiliations:** * Massachusetts General Hospital, Department of Emergency Medicine, Boston, Massachusetts; † Massachusetts General Hospital, Department of Radiology, Boston, Massachusetts

## Abstract

**Introduction:**

Emergency department (ED) crowding is widespread, and can result in care delays, medical errors, increased costs, and decreased patient satisfaction. Simultaneously, while capacity constraints on EDs are worsening, contributing factors such as patient volume and inpatient bed capacity are often outside the influence of ED administrators. Therefore, systems engineering approaches that improve throughput and reduce waste may hold the most readily available gains. Decreasing radiology turnaround times improves ED patient throughput and decreases patient waiting time. We sought to investigate the impact of systems engineering science targeting ED radiology transport delays and determine the most effective techniques.

**Methods:**

This prospective, before-and-after analysis of radiology process flow improvements in an academic hospital ED was exempt from institutional review board review as a quality improvement initiative. We hypothesized that reorganization of radiology transport would improve radiology cycle time and reduce waste. The intervention included systems engineering science-based reorganization of ED radiology transport processes, largely using Lean methodologies, and adding no resources. The primary outcome was average transport time between study order and complete time. All patients presenting between 8/2013–3/2016 and requiring plain film imaging were included. We analyzed electronic medical record data using Microsoft Excel and SAS version 9.4, and we used a two-sample t-test to compare data from the pre- and post-intervention periods.

**Results:**

Following the intervention, average transport time decreased significantly and sustainably. Average radiology transport time was 28.7 ± 4.2 minutes during the three months pre-intervention. It was reduced by 15% in the first three months (4.4 minutes [95% confidence interval [CI] 1.5–7.3]; to 24.3 ± 3.3 min, P=0.021), 19% in the following six months (5.4 minutes, 95% CI [2.7–8.2]; to 23.3 ± 3.5 min, P=0.003), and 26% one year following the intervention (7.4 minutes, 95% CI [4.8–9.9]; to 21.3 ± 3.1 min, P=0.0001). This result was achieved without any additional resources, and demonstrated a continual trend towards improvement. This innovation demonstrates the value of systems engineering science to increase efficiency in ED radiology processes.

**Conclusion:**

In this study, reorganization of the ED radiology transport process using systems engineering science significantly increased process efficiency without additional resource use.

## INTRODUCTION

Emergency department (ED) crowding is a global issue, with myriad and well-documented negative effects on ED patient care measures, including delayed care, medical errors, increased cost, and even mortality.^1^–^10^ In addition, capacity constraints on EDs are worsening and exacerbating the access block for many patients to receive effective, safe, high-quality ED care.[11,12]

In its 2006 report, “Hospital Based Emergency Care: At the Breaking Point,” the Institute of Medicine (IOM) recommended that systems science innovations be used to improve emergency care efficiency and quality.^1^ Yet ED care systems and institutions vary widely, and the ideal solutions are not clear. In addition, patient arrival rates and inpatient bed capacity represent two important factors that are often outside the influence of ED administrators.^13^,^14^ Thus, the most readily available potential solutions reside in systems engineering designed to improve throughput and reduce waste and waits.^15^,^16^

Radiology testing is a frequently used process, and a robust area of potential improvement.^17^ Modeling has shown that decreasing radiology turnaround times improves ED patient throughput and decreases patient waiting time.^18^ While previous research has demonstrated the effectiveness of Lean methodologies in reducing laboratory cycle times,^19^,^20^ further research in radiology and other testing is needed, underscored by the link between ancillary testing and ED length of stay (LOS) and capacity.^21^

### Reducing Waste Through Systems Engineering

Systems engineering science, broadly defined as the study of designing and optimizing systems as a whole, has seen many advances in recent years. And while systems improvement tools are well established in other industries, including auto and service industries, healthcare has lagged behind, and relatively few published studies of its application exist in emergency medicine.^22^–^27^ One example of systems engineering, known as Lean methodology, has excellent potential to improve complex systems of clinical practice while reducing waste.^22^ In brief, Lean is a collection of continuous quality improvement (QI) tools, aimed at the “relentless” pursuit of reducing waste in all forms, and minimizing the non-value added activity within a system. This is achieved through focusing on individual steps in a process in a detailed fashion, usually with a multidisciplinary group of individuals involved in that process. The putative benefits include decreased wait times, increased efficiency, decreased cost, and improved patient care with fewer resources used – in short, being able to do more with less.^22^–^27^ In this way, Lean methodologies frequently incorporate and synergize well with the application of multiple other systems engineering principles, such as demand-capacity matching, queuing theory, and flexible capacity.^22^

### Emergency Radiology as a Microcosm

Emergency medicine and emergency radiology offer somewhat unique systems improvement opportunities as often, increased patient care efficiency both improves quality and reduces cost. This quality improvement manifests through the IOM domains of efficiency, effectiveness, timeliness, and safety. Emergency radiology represents an excellent model of potential improvement, and also an area in which to test approaches with broader ED applicability. ED radiology process flow typically involves multiple steps that must be conducted in series, with frequent potential for delays. In addition, while the extant literature does include some examples of using Lean methodologies to improve radiology process flow, very little has been published about ED radiology specifically.^28^–^32^ In this initiative, systems engineering tools were used to reorganize radiology testing patient flow. We aimed to optimize the plain film radiology testing process, reduce transport time, decrease waste, and measure the effect of the intervention in a robust manner.

**Population Health Research Capsule**

What do we already know about this issue?

Emergency department (ED) crowding has been associated with lower quality of care. Through systems engineering approaches such as Lean methodologies, ED leaders can potentially reduce patient wait times.

What was the research question?

We sought to investigate the impact of systems engineering science targeting ED radiology transport on patient throughput times.

What was the major finding of the study?

The average radiology transport time reduced from 29 minutes to 21 minutes. This was achieved without any additional resources.

How does this improve population health?

This innovation demonstrates the potential value of systems engineering science to increase both patient safety and the patient experience by improving the efficiency of diagnostic testing.

## METHODS

### Study Design

A prospective, before-and after-analysis of radiology process improvements in a hospital ED was used. As a QI initiative using anonymized data only, this study was exempted per institutional review board protocol. All adult patients seen during the study period of 8/2013 to 3/2016 were included. We defined the pre-intervention study period as three months prior to the intervention, 8/2013–11/2013. Implementation of the intervention occurred on 11/9/2013. Post-intervention study periods consisted of three months immediately post intervention (11/2013–2/2014), a separate six months post intervention (3/2014–8/2014), and one full year (3/2015–3/2016), 16 months post intervention. In order to provide a large sample size, we chose a one-year period post intervention to measure the sustainability of observed effects, and to avoid seasonal bias.

### Study Setting and Population

This study was performed in a large, urban, academic, hospital ED with an annual census of approximately 110,000 patient visits. The ED is a Level I trauma center for adult and pediatric patients, and a regional burn center. Approximately 31% of all visits arrive by ambulance, and approximately 26% of patients are admitted to inpatient services. Following patient arrival and registration, patient flow in the ED includes triage, evaluation in a care area, diagnosis and treatment, and disposition. Radiology studies are ordered following initial patient evaluation, and patients are then transported to the ED radiology area when radiology technologists are available to perform the study. The step-by-step testing process is described further below. We included all adult patients seen in the ED who received radiology (plain film) testing.

### Intervention

**Figure 1:**
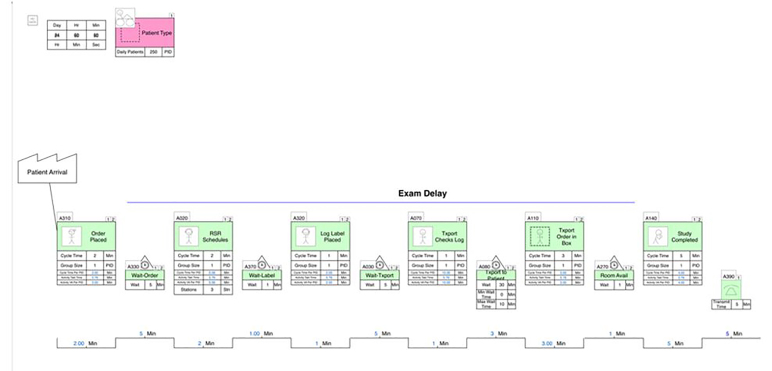
Pre-intervention radiology process flow. Lean value-stream map demonstrating multiple process steps required to achieve plain film radiology testing in the emergency department.

**Figure 2:**
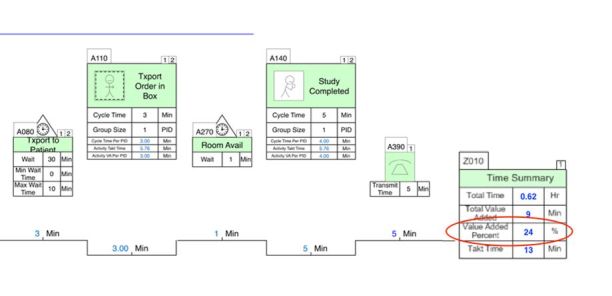
Pre-intervention radiology process flow value-added time summary. Lean value-stream map demonstrating the ability to calculate low value added percent time (24%) of the plain film radiology process, demonstrating opportunity for improvement.

The intervention consisted of a series of process improvement steps based on Lean methodologies, and aimed at reorganizing radiology process flow. The overall aim was to eliminate non-value added waste when possible, with the goal of reducing transport delay. We used a granular, value stream mapping approach to analyze the current state (Figure 1) and identify opportunities to reduce process steps and increase value added activity (Figure 2). In our ED, as in most, a patient is registered, triaged, and then evaluated by a provider. This provider orders diagnostic testing, including plain film imaging when indicated. The order is then scheduled by the radiology scheduling receptionist and populates a queue for the radiology technologists. The patient is then transported to radiology through a number of steps (Figure 1) and then the study is performed. Each of the steps involved in performing plain film radiology following placement of an order were included in the initial process map. In addition, we used supply chain management science, queuing theory, and demand capacity matching to identify other opportunities.

**Figure 3:**
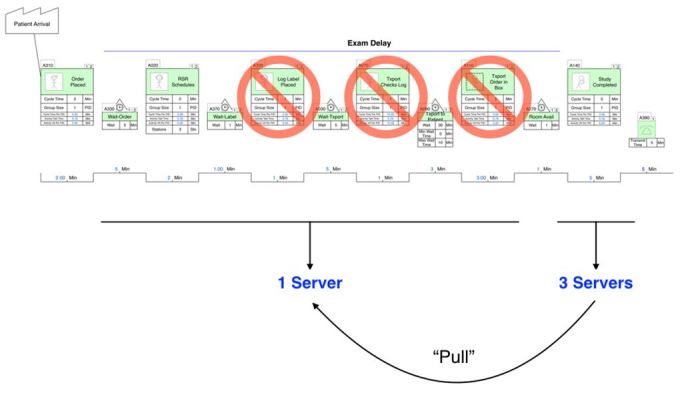
Post Intervention: Systems engineering-based radiology process flow. Lean value-stream map demonstrating opportunity to eliminate process steps and increase efficiency in the process, and minimize the effect of a single server queue.

This resulted in a change to a “pull” system rather than a “push” system, in which patients were actively moved to the subsequent step in their testing by the radiology technologists. In the new design, the technologist-based transport system replaced the single-server transporter, taking advantage of a pooled server approach in which any technologist not currently performing a study would find and transport the next patient in the queue. This resulted in a reduction in the number of process steps and associated bottlenecks (Figure 3).

No additions to staffing or other resources were associated with this intervention. In addition, no other significant operations changes affecting plain film ordering and transport process flow metrics were made in either the ED or ED radiology between the before-and-after measurement periods.

### Methods of Measurement

The primary outcome measure was the ED radiology transport time for plain film testing. This was defined as the time interval in minutes between study order and study start time following patient transport. Data were aggregated on a weekly basis, and we used the average transport delay during each seven-day period in the analysis. The resulting sample sizes for each period were pre intervention (n=12), post intervention three months (n=13), post intervention six months (n=25), and post intervention one year (n=52).

### Data Collection and Analysis

We extracted data from the Radiology Information System (RIS, Boston, MA) during the pre- and post-intervention periods. Testing data were included in the analysis if both time stamps (i.e., study order time and study complete time) were present. No data were specifically excluded from the analysis.

Weekly average delay had a normal-like distribution; therefore, it was summarized using the mean with standard deviation for each period, and each post-intervention period was compared to the pre-intervention period using a two-sample t-test. To address for seasonal effect, we also compared the data from the pre-intervention period (8/4/2013–11/9/2013) to the data from the same period post intervention (8/2/2015–11/7/2015). We used linear regression lines to indicate the trends over different time periods. All analyses were conducted using SAS version 9.4 (SAS Institute, Cary NC), and we considered a two-sided p value of 0.05 or less statistically significant. Statistical review of the study was performed by a biomedical statistician, Yuchiao Chang, Ph.D.

## RESULTS

**Figure 4:**
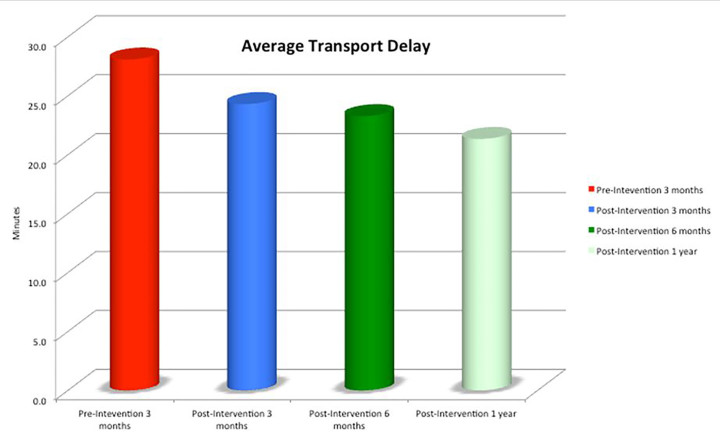
Average radiology transport time 2013–2016. Average radiology transport time following the intervention (minutes), demonstrating a significant trend towards improvement.

**Figure 5:**
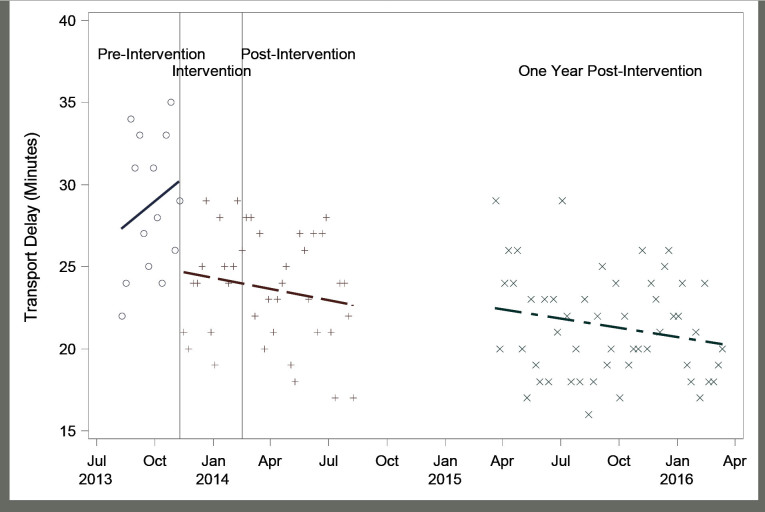
Average radiology transport time. Average radiology transport time (minutes) pre intervention and post intervention three, six, and 12 months following the intervention.

Following the intervention, average transport time decreased significantly and sustainably. Average radiology transport time was 28.7 ± 4.2 minutes during the three months pre intervention. It was reduced by 15% in the first three months (4.4 minutes, 95% CI [1.5–7.3]; to 24.3 ± 3.3 min, P=0.021), 19% in the following six months (5.4 minutes, 95% CI [2.7–8.2]; to 23.3 ± 3.5 min, P=0.003), and 26% one year following the intervention (7.4 minutes, 95% CI [4.8–9.9]; to 21.3 ± 3.1 min, P=0.0001, Figure 4). When comparing the three months pre intervention to the same period post intervention, the average radiology transport time reduced from 28.7 ± 4.2 minutes to 20.6 ± 3.0 minutes (difference 8.1 minutes, 95% CI [5.2–11.0], P<0.0001). This result was achieved without any additional resources, and demonstrated a continual trend towards improvement (Figure 5).

## DISCUSSION

In this before-and-after study, a reorganization of ED radiology process flow significantly and sustainably decreased transport time without additional capabilities or resources. One year following the intervention, transport time was reduced by 24%, or 6.8 minutes. Given the approximately 4,200 plain film visits to ED radiology per month in our ED, there was a reduction of as much as 476 hours of patient wait-time per month, or 5,712 hours per year.

Lean methodologies focus on eliminating non-value added waste within a system.^22^ This includes any and all actions and activities that do not add value to the consumer in question, in this case the patient. In the case of radiology testing, there are a number of areas of potential waste, much of which manifests as waiting for serial process steps.^28^–^32^ As a result, several factors may have contributed to the success of this Lean-based intervention.

First, the job of transporting a patient to radiology was filled by a single individual in the prior state, in what is termed a “single server system.” Based on “queuing theory,” briefly summarized as the science that describes waiting in lines, a system with a single server is by definition the most vulnerable to building a queue when that server’s capacity is overwhelmed by demand. In addition, this effect is magnified when the “arrivals” into that system (in this case, a plain film being ordered for a patient) are variable in their timing. Like most ED patient-care processes, radiology study ordering is highly variable due to varying arrival rates of the patients themselves, varying patient needs and clinical indications, and varying provider practice patterns. 8–32 In addition, our previous single server transport system prioritized transporting patients ordered for computed tomography (CT), magnetic resonance imaging (MRI) or any study ordered from our high acuity area of the ED prior to plain film transports. In a single server system, this further decreased the service capacity from the perspective of the patient awaiting a plain film, and increased wait time.

Further, in the prior system the downstream radiology technician was only responsible for performing and processing images and was resourced with three available plain film radiology bays to accomplish this. This meant that intermittently there was “down time” in this server group, or what is termed “perishable service capacity” in systems engineering (i.e., when one server is idle and that idle time is both wasted and non-recoverable by the system).

Given that radiology technicians seemed to have intermittent available capacity and that their workflow was limited by the single-server system queue just upstream (or “bottleneck”), it is not surprising that an intervention aimed at eliminating this bottleneck by asking the technicians to help with transport when idle, and “pull” patients into testing, was successful. In systems engineering, this combination of tasks within a group of servers is referred to as “pooling servers,” described as the process by which multiple servers are asked to bring together, or pool, their task lists and workflow. This has the putative benefit of allowing multiple servers to be available and balancing server capacity with the demand for that service. However, it is worth mentioning that asking a downstream server (e.g., radiology technician) to perform an upstream task is not without risk of what is termed “shifting bottlenecks.” For example, if the added task reduces their ability to perform the downstream task (e.g., performing the plain film), and thus this task forms a queue and associated delay, then this would become the new bottleneck for the system.

Regarding the cost vs. benefit implications of this project, the operational and efficiency benefit gained from this intervention appeared to outweigh the minimal resources (i.e., ED administrator time and effort) used to carry out these systems changes. No resources were added during the intervention, and yet measurable and sustainable reductions in radiology transport times were noted. This innovation demonstrates the potential value of systems engineering science to increase efficiency in ED radiology processes, and increase system capacity, a benefit of Lean methodologies that has been demonstrated in other studies.^31^–^33^ Additionally, this work may inform radiology staffing decisions and workflow, and underscores the value of current state mapping and analysis, demand-capacity matching, and pooled-server resource use while adapting to changing workflows.^34^ While other studies have demonstrated the value of systems engineering approaches in optimizing ED processes, these results underscore that significant opportunity to improve on key performance indicators and broaden the literature and experience in ED radiology remains.^33^–^38^

Finally, as ED administrators increasingly focus on ED patient experience, interventions that reduce patient waits while also improving the efficiency of diagnostic testing may represent a valuable approach for emergency medicine and emergency radiology administrators to achieve the win/win of enhancing patient care and experience simultaneously.

## LIMITATIONS

As with any before-and-after study, while the change demonstrated in the observed outcomes may be correlated to the intervention, this does not prove causality. We could not fully exclude other contributing factors, such as subtle differences in the patient population studied or in individual productivity; however, it is unlikely that these factors played a significant role in the results. In addition, given that our unit of measure was weekly, we cannot fully exclude daily changes in volume as contributing to the effect, although given the duration of the study it is unlikely that this effect was due to daily changes in volume. Although we cannot fully exclude the Hawthorne effect from playing a role, its effect, if any, was likely limited due to the duration of the study and the fact that the staff was not aware of the focus on this metric. In addition, no other significant operations changes affecting plain film ordering and transport processes were identified in either the ED or ED radiology between the before-and-after measurement periods, and other potential contributors including testing volume did not change significantly during the period studied. The pre-intervention period of three months is also somewhat short but was chosen due to data availability limitations. Given the large number of studies performed during those three months, we believe this period had adequate sample size, and we compared a similar period post intervention to confirm a lack of seasonal effect. In addition, there was no change in the cycle time of the other radiology tests (e.g., CT, MRI) before and after the intervention. Thus, it is likely that the intervention was associated with the outcome measured.

The study was performed at a single center, potentially limiting generalizability to other EDs, especially those with markedly different radiology process flows and/or demographics. However, given that systems engineering tools are broadly applicable by definition, we anticipate our findings should be of interest to most ED and ED radiology administrators.

Finally, our study design did not permit measuring any increased radiology productivity as a result of these improvements, nor was it able to correlate decreased radiology turnaround time with decreased ED length of stay or other ED metrics such as left without being seen. While it may be assumed that a more efficient system may be more productive and less wasteful, this cannot be proven by our study.

## CONCLUSION

In this study, reorganization of the ED radiology transport process using systems engineering science measurably increased process efficiency without additional resource use.
